# Point-of-care echocardiography of the right heart improves acute heart failure risk stratification for low-risk patients: The REED-AHF prospective study

**DOI:** 10.1111/acem.14589

**Published:** 2022-09-26

**Authors:** Nicholas E. Harrison, Mark J. Favot, Laura Gowland, Jacob Lenning, Sarah Henry, Sushane Gupta, Aiden Abidov, Phillip Levy, Robert Ehrman

**Affiliations:** 1 Indiana University School of Medicine, Indianapolis, Indiana, USA; 2 Wayne State University, Detroit, Michigan, USA

**Keywords:** cardiology, echocardiography, emergency department, emergency medicine, heart failure, point-of-care ultrasound, risk stratification

## Abstract

**Objectives:**

Validated acute heart failure (AHF) clinical decision instruments (CDI) insufficiently identify low-risk patients meriting consideration of outpatient treatment. While pilot data show that tricuspid annulus plane systolic excursion (TAPSE) is associated with adverse events, no AHF CDI currently incorporates point-of-care echocardiography (POCecho). We evaluated whether TAPSE adds incremental risk stratification value to an existing CDI.

**Methods:**

Prospectively enrolled patients at two urban-academic EDs had POCechos obtained before or <1 h after first intravenous diuresis, positive pressure ventilation, and/or nitroglycerin. STEMI and cardiogenic shock were excluded. AHF diagnosis was adjudicated by double-blind expert review. TAPSE, with an a priori cutoff of ≥17 mm, was our primary measure. Secondary measures included eight additional right heart and six left heart POCecho parameters. STRATIFY is a validated CDI predicting 30-day death/cardiopulmonary resuscitation, mechanical cardiac support, intubation, new/emergent dialysis, and acute myocardial infarction or coronary revascularization in ED AHF patients. Full (STRATIFY + POCecho variable) and reduced (STRATIFY alone) logistic regression models were fit to calculate adjusted odds ratios (aOR), category-free net reclassification index (NRI_cont_), ΔSensitivity (NRI_events_), and ΔSpecificity (NRI_nonevents_). Random forest assessed variable importance. To benchmark risk prediction to standard of care, ΔSensitivity and ΔSpecificity were evaluated at risk thresholds more conservative/lower than the actual outcome rate in discharged patients.

**Results:**

A total of 84/120 enrolled patients met inclusion and diagnostic adjudication criteria. Nineteen percent experiencing the primary outcome had higher STRATIFY scores compared to those event free (233 vs. 212, *p* = 0.009). Five right heart (TAPSE, TAPSE/PASP, TAPSE/RVDD, RV-FAC, fwRVLS) and no left heart measures improved prediction (*p* < 0.05) adjusted for STRATIFY. Right heart measures also had higher variable importance. TAPSE ≥ 17 mm plus STRATIFY improved prediction versus STRATIFY alone (aOR 0.24, 95% confidence interval [CI] 0.06–0.91; NRI_cont_ 0.71, 95% CI 0.22–1.19), and specificity improved by 6%–32% (*p* < 0.05) at risk thresholds more conservative than the standard-of-care benchmark without missing any additional events.

**Conclusions:**

TAPSE increased detection of low-risk AHF patients, after use of a validated CDI, at risk thresholds more conservative than standard of care.

## INTRODUCTION

Acute heart failure (AHF) accounts for 1 million emergency department (ED) visits annually^1^ and presents significant dilemmas for risk stratification and ED disposition.^2^ Over 80% of AHF patients treated in United States EDs are admitted to the hospital, but as many as half may be low enough risk to avoid admission.^3–6^ The forthcoming 2022 update to the American College of Emergency Physicians (ACEP) clinical policy on AHF^7^ identified three validated clinical decision instruments (CDI)^8–10^ that are useful for risk-stratification: the STRATIFY risk score,^8,11^ the Emergency Heart Failure Mortality Risk Grade (EHMRG),^9,12^ and the Ottawa Heart Failure Risk Scale (OHFRS).^10,13^ While all three have validation test characteristics that exceed published performance of emergency physician (EP)-estimated risk^9^ and actual disposition decisions,^12^ none were deemed sufficient to direct AHF disposition on their own. Recent multicenter studies have shown that ED discharged AHF patients experience 30-day rates of death (3.8%)^14^ and serious adverse events (7.1%) in STRATIFY’s composite outcome^6^ much greater than risk thresholds that EPs report as acceptable.^15^ Nonetheless, in one study comparing EHMRG^9^ to EP-estimated risk, not only did EHRMG perform significantly better but EPs frequently misclassified the highest risk patients as low risk (and vice versa). Given this, recent SAEM-endorsed expert consensus guidelines have identified “safely transitioning a larger proportion of patients to the outpatient setting”^16^ and “prediction of low risk”^5^ as critical needs in AHF clinical research.

In a previous pilot study we identified that tricuspid annulus plane systolic excursion (TAPSE) on EP-performed point-of-care echocardiography (POCecho) is associated with short-term adverse AHF events.^17^ This compares to numerous studies of HF patients outside the ED setting, where TAPSE and other measures of right ventricular dysfunction (RVD) and/or pulmonary hypertension (PH) predict adverse outcomes,^18–29^ often more strongly than LV dysfunction^30–42^ or biomarkers.^37,42–46^ Conversely, there is a stark absence of prior ED risk stratification studies that incorporate or consider echocardiography measures. Nevertheless, TAPSE^17,38,47–49^ and other right heart measures^38,50^ have been used by EPs with high interobserver reliability in the risk stratification of pulmonary embolus, suggesting feasibility for AHF risk stratification.

The Right ventricular Echocardiography in ED AHF (REED-AHF) study was a two-center prospective cohort^38^ whose primary objective was to evaluate if ED TAPSE had additive and incremental predictive value for 30-day serious adverse events when used in conjunction with a validated and ACEP-endorsed CDI such as STRATIFY. We hypothesized that TAPSE, at an a priori ≥ 17-mm cutoff from our pilot study,^17^ would improve risk stratification for STRATIFY’s 30-day composite of serious adverse AHF events compared to STRATIFY alone. Secondarily, we sought to characterize the incremental value of 14 additional POCecho measures for the same purpose and evaluate how these alternative measures compare to TAPSE by variable importance and predictive value after adjusting for STRATIFY.

## METHODS

The primary methods, protocol, design, and patient cohort of REED-AHF have been described previously.^38^ REED-AHF was funded by an investigator-initiated grant (NH) from the Blue Cross Blue Shield Foundation of Michigan and approved by the Wayne State University Institutional Review Board. This report describes the primary aim of the study. This report has been prepared to adhere to the STROBE guidelines.^51^

### Study design, setting, and population

REED-AHF was a prospective multicenter cohort study at two urban EDs with 80,000–100,000 annual visits each. Detroit Receiving Hospital is an urban-academic referral hospital and Level I trauma center; Sinai-Grace Hospital is an urban-community Level II trauma center. Both sites host 3-year emergency medicine (EM) residencies.

Patients were enrolled from September 2019 to February 2020 and November 2020 through March 2021. Enrollment was paused during the intervening 8 months per institutional policies regarding clinical research during the early phase of the COVID-19 pandemic. Patients were screened 7 days per week by trained research staff and study personnel; however, enrollment was limited to times when a study sonographer was available.^38^ Despite this, 25% of patients were enrolled between the hours of 5 p.m. and 8 a.m. and 18% on weekends.^38^

Full inclusion, exclusion, and diagnostic adjudication criteria have been described previously.^38^ Patients with cardiogenic shock, STEMI, tachydysrhythmia requiring intravenous (IV) rate control and those deemed not to have AHF by the EP at disposition were excluded. Patients had to receive a POCecho and lung ultrasound (LUS) within <1 h after, or any time before, first initiation of any qualifying AHF treatment: IV loop diuretics, SL or IV nitroglycerin, noninvasive positive pressure ventilation (NIPPV), and/or intubation. Two experts (AA, MF) blinded to study outcomes adjudicated the ED diagnosis of AHF.^38^

### Study protocol

The POCecho protocol, performed using a Vivid q ultrasound system, has been described previously.^38^ A majority of POCechos were performed by a single EP sonographer (NH) without POCUS fellowship training, with fewer performed by a POCUS-trained co-investigator (RE or MF), PGY-2 EM resident (JL), or research cardiac sonographer (LG). Measurements were made first on the POC platform. A second offline measurement by a different investigator^38^ blinded to the POC measures and clinical outcomes was also performed, to allow calculation of intraclass correlation (ICC) for all measures. All POCecho measures (see list of specific measures below) except RVOT acceleration time showed moderate–high inter-rater reliability (ICC > 0.7) between POC and offline analysis. Per protocol,^38^ RVOT acceleration time was then overread by a testamur (RE) of the examination of special competence in adult echocardiography administered by the National Board of Echocardiography (NBE). Another NBE testamur (MF) performed blinded overreads of the free-wall right ventricular longitudinal strain (fwRVLS) measure on account of this being a more advanced echo measure, yielding ICC > 0.7.

### Primary clinical risk measure: the STRATIFY risk score

Demographic and clinical variables were collected prospectively at enrollment, throughout hospitalization, and through 90-day follow-up as previously described.^38^ The primary ED clinical measure of interest was the STRATIFY score,^8^ and its component variables of age, BMI, brain natriuretic peptide, blood urea nitrogen, dialysis history, diastolic blood pressure, sodium, ED supplemental oxygen requirement, outpatient ACE inhibitor, QRS prolongation on electrocardiogram, respiratory rate, oxygen saturation, and cardiac troponin I.

STRATIFY was chosen as the primary clinical risk measure for a few reasons. First, as cited above, it is one of three CDIs endorsed by ACEP to be helpful in risk stratification.^7^ Second, compared to EHMRG and OHFRS, it contains the largest number and greatest diversity of clinical variables which an EP may use and have access to in clinical decision making. Third, STRATIFY was derived to predict a hierarchical composite of several serious adverse AHF events which all have clinical significance (described below).^8^

### Primary clinical outcome

The primary clinical outcome was the 30-day composite of serious adverse AHF events which STRATIFY was derived^8^ and then validated^11^ to predict. The composite includes death/CPR, mechanical cardiac support, intubation, new or emergent dialysis, and/or acute myocardial infarction (AMI)/percutaneous coronary intervention (PCI)/coronary artery bypass grafting (CABG). Previous validation^11^ showed STRATIFY to predict this outcome with >0.7 area under the receiver operating characteristic curve (AUROC).

Outcomes were adjudicated by telephone follow-up, supplemented by multiple blinded abstractors reviewing electronic medical record data and health information exchange data for the state of Michigan as previously described.^38^ AMI was defined based on the Fourth Universal Definition of MI,^52^ adjudicated by multiple blinded raters.

### Data analysis

All statistical analyses were performed in the R language (R Studio 2021.09.1). Patient characteristics were stratified by those with versus without the primary outcome, and echocardiography parameters by outcome and ED disposition, to calculate descriptive statistics using the Wilcoxon rank-sum test for continuous variables and Fisher’s exact test for categorical variables (α = 0.05).

#### Primary echo measure

Based on our pilot study which showed association between adverse events and TAPSE < 17mm^17^ and other published data in non-ED settings, our primary measure of interest was TAPSE, which was analyzed as a continuous variable with an a priori binary cutoff of ≥17 mm. To provide 80% power to detect an adjusted odds ratio (aOR) of 0.30 at this cutoff for prediction of the primary outcome, 111 patients were needed.

#### Secondary POCecho measures for comparison and exploratory analysis

Additional right heart measures included fwRVLS, RV-LV ratio, pulmonary artery systolic pressure (PASP) by tricuspid regurgitant jet velocity (i.e., which is also the right ventricular systolic pressure [RVSP] under most scenarios}, TAPSE/PASP ratio, TAPSE/right ventricular diastolic diameter ratio (TAPSE/RVDD), RV fractional area change (FAC), pulmonary vascular resistance, and RVOT acceleration time. Left heart measures included left ventricular ejection fraction (LVEF), mitral E/A ratio, septal E’, lateral E’, E/E’ ratio, and LV global longitudinal strain (LVGLS) by speckle tracking.

Exploratory analyses were conducted with two goals. First, we explored the possibility that measures besides TAPSE may have incremental value in risk stratification, since few descriptions of any POCecho measure exist in ED-based AHF risk stratification literature. Second, the other measures served as indirect comparators to TAPSE in evaluating our primary hypothesis. We approached comparison in two ways: (1) evaluating relative variable importance of all POCecho measures with STRATIFY in a random forest model and (2) modeling each POCecho variable in the same way as TAPSE in full (POCecho measure plus STRATIFY) and reduced (STRATIFY only) logistic regression models for measures of incremental improvement in prediction (logistic regression methods, below). Secondary POCecho measures were also analyzed as continuous variables and using a priori binary cutoffs corresponding to the American Society of Echocardiography guidelines^53–55^ or, in the absence of a guideline-recommended value, prior AHF literature from settings outside the ED.^17,25,26,56,57^

#### Importance by random forest

The random forest model for assessing variable importance was run under the following specifications: (1) all 15 echo variables including TAPSE and the STRATIFY score were able to be incorporated into classification trees; (2) no more than two total variables (STRATIFY, plus one POCecho variable) were allowed to be sampled in a single classification tree (to prevent overfitting from any single model, e.g. incorporating three or more variables); and (3) 10,000 trees were grown for prediction of the clinical primary outcome using the randomForest package in R, with importance (mean decrease in Gini) calculated and plotted. Random forest utilized in this way aids in selection of independent variables (features) in the context of high dimensionality by calculating the importance of each variable in predicting the outcome of interest, of which mean decrease in Gini is a common importance measure.^58^ The expected outcome was that STRATIFY would rise to the top as the most important overall variable, followed by the POCecho variables of importance after accounting for STRATIFY.

#### Logistic regression and measures of incremental predictive value/improvement

##### Risk thresholds

Reduced (STRATIFY score alone) and full (POCecho measure plus STRATIFY) logistic regression models were fit^59^ for the primary outcome to calculate unadjusted odds ratios (OR), aOR, and category-free (i.e., continuous) net reclassification index (NRI_cont_). NRI measures the degree by which addition of a single variable to an existing prediction model improves the classification of that model, focused on the observations which are reclassified to a different prediction category by the added predictor, and specifically whether or not those reclassifications resulted in fewer or greater correct classifications overall (i.e., net).^60^ The NRI_cont_ assesses classification across all risk thresholds, while the categorical NRI assesses reclassification at specific risk cutoffs.^60^ Variance inflation factor (VIF) was calculated to assess models for multicollinearity; all VIF were < 3. Models were internally validated using the bootstrap to assess optimism and produce bias-corrected estimates of AUROC,^59^ so that reported estimates of performance are lower (more conservative) than the observed estimates, to account for the fact that new models tend to perform worse on external validation. Categorical NRI for various risk-thresholds were calculated separately for events (NRI_events_) and nonevents (NRI_nonevents_) by methods previously described^60^ to enhance clinical utility and interpretability. Calculated this way, NRI_nonevents_ = ΔSpecificity and NRI_events_ = ΔSensitivity—i.e., the improvement or worsening in sensitivity and specificity in the standard risk stratification (i.e., STRATIFY score) after adding a novel risk marker (i.e., POCecho variable such as TAPSE)^60^; 95% confidence intervals (95%CI) were calculated and reported.

#### Benchmarking to clinical standard of care decision making

There is no currently agreed upon “acceptable” miss rate for serious adverse events at which patients should be considered for outpatient treatment of AHF after ED treatment. To account for this, we benchmarked categorical risk prediction and reclassification to standard-of-care risk stratification. Model predicted risk thresholds (i.e., miss rates) were chosen to be below the true/observed rate of the primary outcome among patients actually discharged from the ED by standard EP decision making. Thus, measures of categorical reclassification (NRI_events_/ΔSensitivity and NRI_nonevents_/ΔSpecificity) were calculated at thresholds that would ensure that the model’s “miss rate” was more conservative than the standard-of-care miss rate. The rationale of this approach was that even if the exact value of an “acceptable miss rate” is debated, most EPs would likely agree that a model which misses fewer events than the standard of care is potentially useful, particularly if it can also increase identification of low risk (i.e., improved NRI_nonevents_/ΔSpecificity) without adding missed events (i.e., no decline in sensitivity).

To increase precision in estimating the benchmark standard-of-care miss rate, we averaged the rate of events in discharged patients in REED-AHF^38^ with the rate observed in CLEAR-AHF, a recent 257 prospective patient cohort from the same institution.^6^ CLEAR-AHF^6^ had similar adjudication of AHF diagnosis, adjudicated outcomes for the STRATIFY risk score, and similar inclusion criteria as REED-AHF, but was larger than REED-AHF and had slightly lower outcome rates.^38^ Among 341 patients in both cohorts, 8.3% of discharged patients experienced the primary outcome compared to 13.6% among admitted or observation patients.^6,38^ Thus, 8% was chosen as the higher risk threshold to be evaluated (i.e., the least conservative integer risk threshold to still be more conservative than usual care). A low-end risk threshold of 3% was chosen to match the smallest risk-threshold for which STRATIFY was derived and validated.^8^

#### Sensitivity analysis: comparison to clinical features not captured by STRATIFY

Although STRATIFY was chosen as the primary baseline risk measure in part because of how many different clinical variables it incorporates, some important decision-making factors for EP risk stratification of AHF nevertheless are absent from the risk score. We analyzed several clinical factors not included in STRATIFY that could hypothetically add predictive value. First, past history of chronic obstructive pulmonary disease (COPD), PH, ED systolic blood pressure (SBP), pre-ED adherence to guideline-directed medical therapy, and sex were chosen a priori as patient-level factors that could be associated with both TAPSE and outcomes while not being incorporated in STRATIFY. Several treatment-level variables were chosen based on being possible therapeutic indications why a patient would not be a candidate for outpatient treatment (i.e., even if low risk for adverse events), including total ED IV furosemide dose, ED NIPPV, ED IV nitroglycerin, and any new or increased oxygen requirement during the ED visit compared to baseline. Finally, EP disposition decisions of discharge versus admission and ICU versus another level of care were included to assess actual clinical decision-making compared to the POCecho variables. EPs were blinded to all POCecho data during the study, including when selecting disposition.

Each of the variables here was tested for NRI_cont_ when added to STRATIFY by similar methods as described above for the echocardiography variables in sensitivity analyses. The hypothesis of these sensitivity analyses was that significant improvement in classification by any of these variables compared to STRATIFY alone would diminish the importance of any risk stratification benefit observed for a POCecho variable, since these clinical variables are more easily obtained through history and medical record review than a POCecho, are commonly found in current AHF risk stratification literature and ED-based CDIs, and/or inherently reflect the standard-of-care in EP decision-making.

## RESULTS

### Enrollment and time to POCecho

A total of 120 patients met initial inclusion criteria, with POCechos obtained at a median of −75 min (interquartile range [IQR] −131 to +13) before first IV loop diuretic, +33 min (+6 to +59) after initiation of PPV, −21 min (−49 to +23) before first IV/SL nitroglycerin, +33 min (−234 to +162) after first supplemental O_2_ initiation, and +102 min (+47 to +177) after ED arrival. A CONSORT diagram with enrollment details has been previously published.^38^ After exclusions for EP diagnosis at disposition of “not-AHF” (*n* = 11), failure to obtain consent from the patient or a representative within 24 hours of the POCecho (*n* = 20), and expert-adjudicated diagnoses of not-AHF (*n* = 5), 84 patients remained for analysis.

### Descriptive clinical and echocardiographic characteristics

Description for selected clinical and POCecho measures are presented in Tables 1 and 2, respectively. Median (IQR) age was 62 (54–70) years, LVEF 28% (19%–38%), TAPSE 17 (13–23) mm, SBP 153 (132–173) mm Hg, and STRATIFY score 215 (198–233). Sixty percent were male (*n* = 50), 18% received NIPPV (*n* = 15), 3.6% (*n* = 3) were admitted to the ICU, 14% (*n* = 12) placed in outpatient observation, and 9.5% (*n* = 8) discharged from the ED.

Nineteen percent of patients (*n* = 16) had one or more serious adverse AHF events in the composite primary outcome at 30 days, including three (3.6%) deaths. Experiencing the primary outcome was associated with (Table 1) total STRATIFY score and the STRATIFY components of dialysis history and sodium concentration.

Among POCecho variables (Table 2), four right heart measures and no left heart measures were associated with the primary outcome: TAPSE (*p* = 0.011), TAPSE/PASP (*p* = 0.035), TAPSE/RVDD (*p* = 0.011), and fwRVLS (*p* = 0.034). There was no difference in TAPSE between admitted versus discharged patients (Table 2) to suggest unblinding of the treating EPs to the POCecho results.

### Category-free reclassification

TAPSE improved category-free reclassification of events and nonevents compared to STRATIFY alone (NRI_cont_ +0.63 [95% CI +0.08 to +1.18]), as did TAPSE/RVDD (+0.67 [+0.18 to +1.16]) and fwRVLS (+0.49 [+0.01 to +0.98]). Significant reclassification by NRI_cont_ was not observed for other POCecho variables or any of the clinical variables of interest including past history of COPD, history of PH, new or increasing supplemental O_2_ requirement, ED IV loop diuretic dose, ED NIPPV or intubation, ED IV nitroglycerin, or disposition decisions of ICU or discharge home (Table 3).

### Categorical reclassification and improvement in test characteristics at clinically benchmarked risk thresholds

**FIGURE 1:**
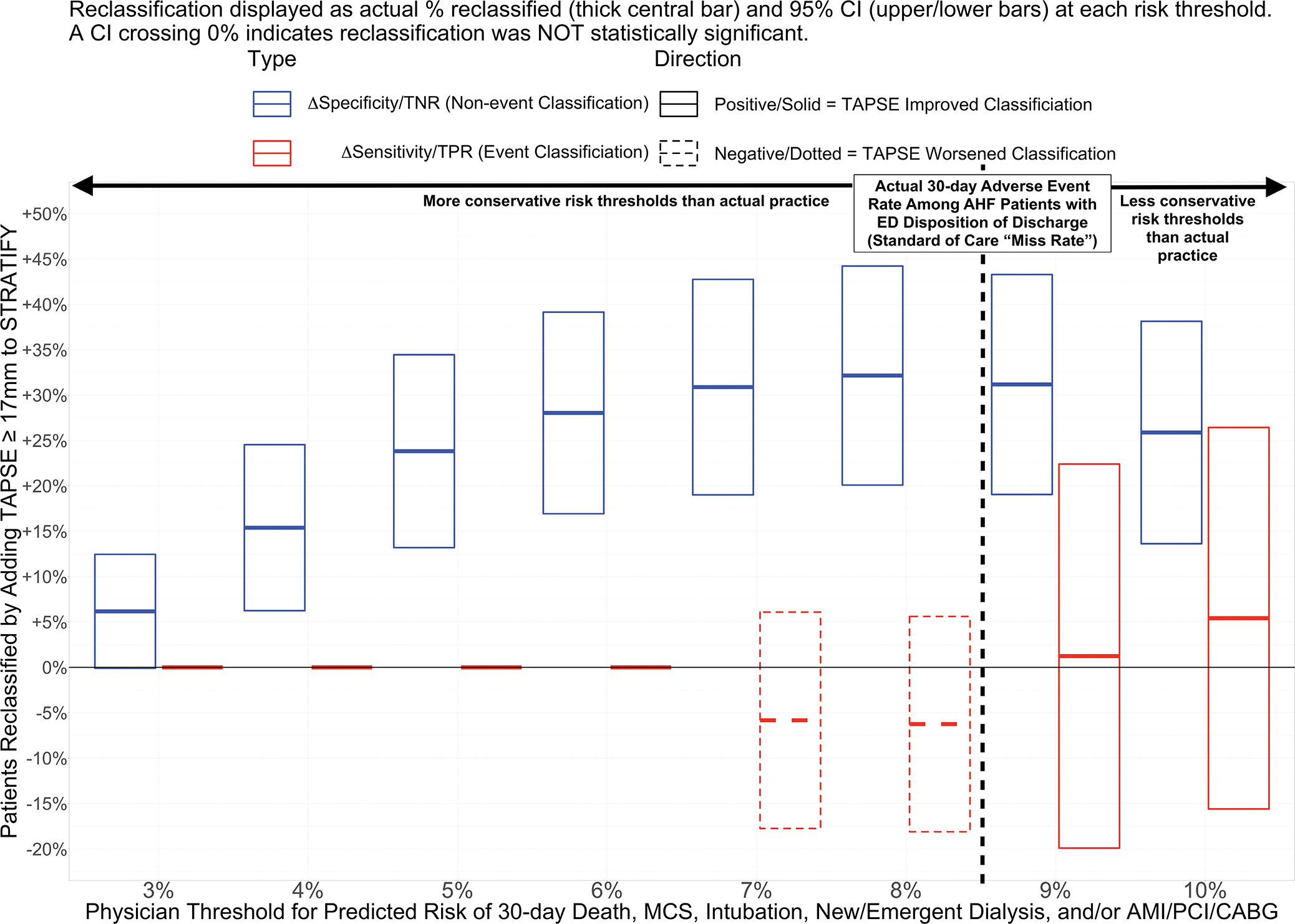
Change in sensitivity (NRI-Events) and specificity (NRI-Nonevents) when TAPSE is added to STRATIFY versus STRATIFY alone, for various thresholds of 30-day risk for serious adverse heart failure events. When added to the STRATIFY risk score, TAPSE ≥ 17 mm increased specificity (TNR/NRI_nonevents_, i.e., correct identification of patients without a 30-day event) without any significant change in sensitivity (i.e., no change in missed events) for risk thresholds benchmarked to the actual 30-day outcome rate among discharged patients. If the use of TAPSE + STRATIFY were used conservatively (i.e., only at risk thresholds more conservative than the adverse event rate occurring under standard-of-care EP disposition decision making) there still would have been a significant increase in the number of low-risk patients identified without significant change in missed cases. AMI, acute myocardial infarction; CABG, coronary artery bypass graft; NRI, net reclassification index; MCS, mechanical cardiac support; PCI, percutaneous coronary intervention; TNR, true-negative rate = NRI_nonevents_ = Δspecificity; TPR, true-positive rate = NRIevents = Δ sensitivity; TAPSE, tricuspid plane systolic excursion.

ED treatments and disposition decisions were similar between events and nonevents (Table 1). Figure 1 shows the ΔSensitivity (NRI_events_) and ΔSpecificity (NRI_nonevents_) obtained when TAPSE at the a priori cutoff of ≥17 mm was added to STRATIFY. At every risk threshold less (i.e., more conservative) than the 8.3% benchmark (standard-of-care event rate among discharged patients), TAPSE ≥ 17 mm significantly increased specificity without changing sensitivity. At the 8% cutoff, a peak 32% (95% CI 20%–44%) of event-free patients at 30 days would have been appropriately reclassified from high risk (i.e., by STRATIFY alone) to low risk (by STRATIFY and TAPSE ≥ 17 mm). At an even more conservative 4% predicted risk cutoff, 15% (6%–24%) of patients incorrectly classified as high risk by STRATIFY alone would have been appropriately reclassified to low risk. Figure S2 shows categorical reclassification for all of the 15 POCecho variables side-by-side. TAPSE, the TAPSE/PASP and TAPSE/RVDD ratios, and fwRVLS stood out across multiple cutoffs for increasing specificity beyond STRATIFY alone without sacrificing sensitivity.

### Echocardiographic measure importance

**FIGURE 2:**
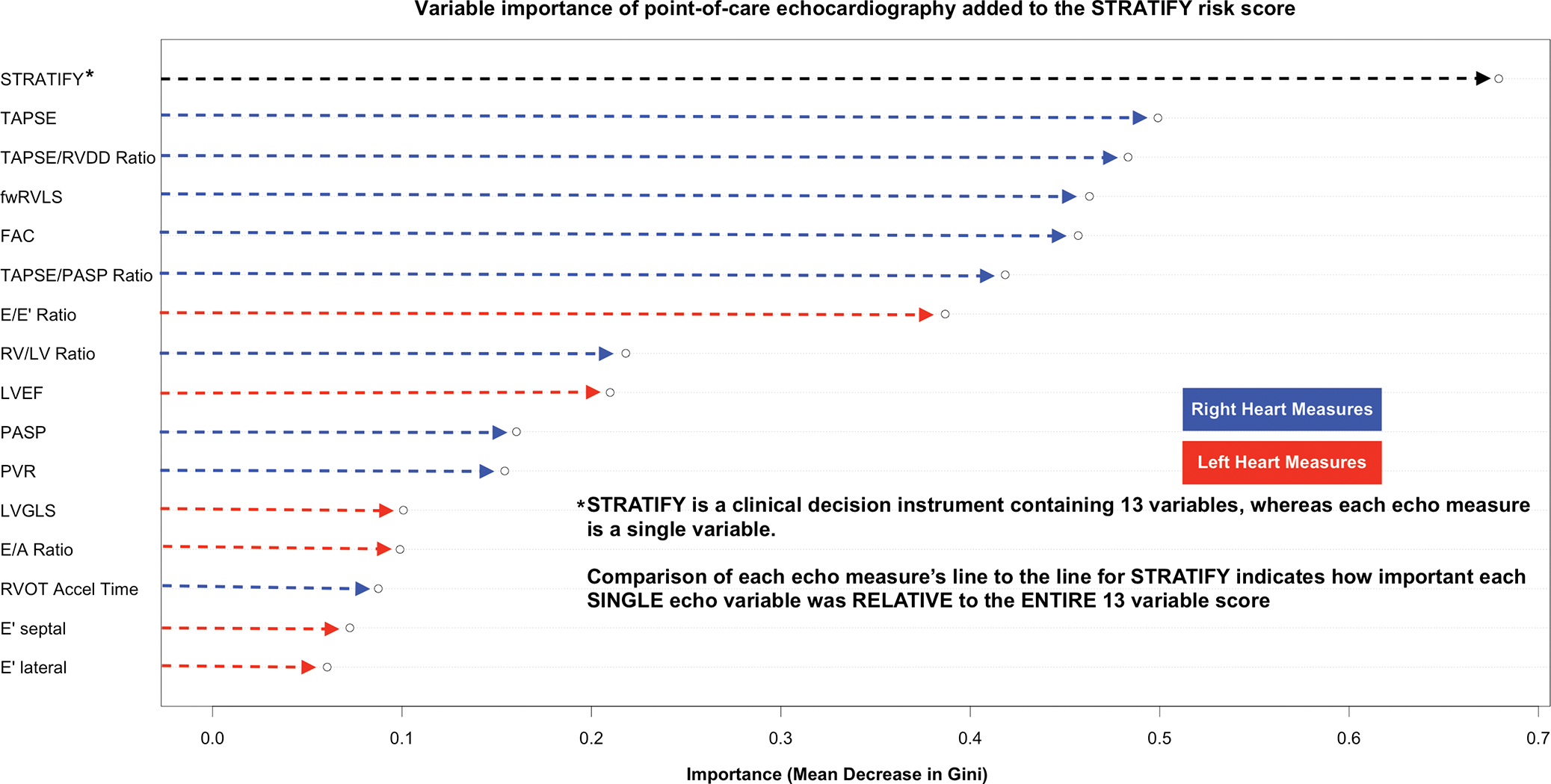
Relative importance of echocardiographic variables for predicting 30-day serious adverse heart failure events in conjunction with the STRATIFY decision instrument importance on random forest of each echocardiography variable in predicting the primary outcome of the STRATIFY decision score* after adjusting for the STRATIFY score itself. Eight of the 10 variables which added the most risk prediction to STRATIFY were right heart measures. TAPSE, the primary measure of interest, was the most important echocardiography variable. As a single variable, TAPSE was >70% as important as the entire 13 variable STRATIFY score in predicting STRATIFY’s outcome. *30-day death, CPR, mechanical cardiac support, intubation, new or emergent dialysis, acute myocardial infarction, and/or coronary revascularization. FAC, RV fractional area change; fwRVLS, free-wall RV longitudinal strain; LV, left ventricle; LVEF, left ventricular ejection fraction; LVGLS, LV global longitudinal strain; PASP, pulmonary artery systolic pressure; PVR, pulmonary vascular resistance; PW, pulsed wave; RV, right ventricle; RVDD, right ventricular diastolic diameter; RVOT, right ventricular outflow tract; TAPSE, tricuspid annular plane systolic excursion.

Figure 2 presents variable importance for each POCecho measure in predicting the primary outcome. TAPSE was the most important POCecho measure. Despite being only a single variable, TAPSE was nevertheless almost three-fourths as important on its own as the entire 13-variable STRATIFY score. The five most important POCecho variables were right heart measures. E/e’ was the most important left heart measure, followed by LVEF and LVGLS.

### Echo measures adjusted for STRATIFY

Figure S1 presents ORs and the STRATIFY aOR’s for each POCecho measure and the primary outcome, presented as continuous (OR/aOR per change in IQR) and binary variables. TAPSE had an aOR of 0.32 (95% CI 0.12–0.87) as a continuous variable and 0.24 (0.06–0.91) at the ≥17 mm a priori cutoff. Internally validated (bias-corrected) AUROC was 0.756 for TAPSE plus STRATIFY versus 0.706 in STRATIFY alone.

Four additional right heart variables were associated with the outcome after adjusting for STRATIFY: TAPSE/RVDD ratio (aOR 0.35 [0.13–0.94]), TAPSE/PASP ratio (0.38 [0.15–0.96]), RV-FAC (0.41 [0.17–0.94]), and fwRVLS (0.31 [0.11–0.9]). RV-LV ratio < 1 (but not as a continuous variable) was also associated with the primary outcome after adjustment (0.24 [0.06–0.78]). No left heart measures were associated with the primary outcome.

## DISCUSSION

In this study, we prospectively validated pilot data suggesting that TAPSE improves risk stratification of AHF patients in the ED for clinically relevant adverse AHF events. We highlight four major findings. First, TAPSE improved risk classification beyond the previously validated^11^ and ACEP-endorsed^7^ STRATIFY CDI by multiple measures: NRI_cont_, aOR, and the ability to identify low-risk AHF patients (i.e., higher NRI_nonevent_/ΔSpecificity) without increasing “missed” high-risk patients (i.e., no change in NRI_event_/ΔSensitivity). Second, TAPSE and other right-heart measures showed greater importance (Figure 2), effect size on STRATIFY adjusted odds (Figure S1), reclassification (Table 3), and prediction of low-risk (Figures 1 and S2) than left heart measures. Third, TAPSE improved classification even where other clinical factors not in the STRATIFY score did not (e.g., ED disposition, COPD, PH history, supplemental oxygen requirement, IV diuretic dose, NIPPV, etc.; Table 3). Fourth, improved identification of low-risk patients with TAPSE (Figure 1) was observed even when benchmarked to ensure that TAPSE miss rates were more conservative than real EPs’ actual disposition decisions when blinded to the POCecho results.

This is the first and most comprehensive prospective report on the utility of POCecho to aid in AHF risk stratification in the ED. Sax et al.^62^ recently described the prognostic utility of historical LVEF in AHF in a large retrospective study, which we believe to be one of the few reports describing any echocardiographic variable in ED risk-stratification, whether POC or not. In addition to REED-AHF, other POCecho studies such as one by Favot et al.^63^ and a previously reported secondary aim of REED-AHF^38^ have described the utility of longitudinal changes from ED arrival to inpatient timepoints, including with TAPSE.^38^ Nevertheless, among the six or more described CDIs for ED risk stratification of AHF, none incorporate echocardiographic variables.^64^ This is despite copious literature describing the utility of right heart echocardiography measures for heart failure risk prediction outside of the ED setting.^6,18–37,39–46^

That echocardiography has so far been excluded from AHF risk stratification in the ED is surprising. Namely, being able to visualize the main organ affected in AHF to assess severity of presentation has clear face validity. Moreover, POCecho measures including TAPSE have been used with reliability and utility by EPs for risk stratification of other conditions like pulmonary embolism (PE).^47,48,50^ Nevertheless, prior literature on the appropriate use of POC ultrasound in AHF for ED patients is highly limited to diagnosis and assessment of volume status^65^ but not risk prediction. LUS, but not POCecho, has also been described for the latter purpose including the recent BLUSHED-AHF trial^66^ and the previous publication from REED-AHF.^38^ Concurrently, even the three strongest CDIs^8–10^ described for ED risk stratification, two of which have been documented to outperform EP gestalt,^9,10^ are viewed by ACEP as potentially useful but nevertheless not strong enough to guide disposition on their own.^7^ With the existence of at least six different ED CDIs for AHF risk prediction utilizing a large overlap of the same clinical variables,^64^ echocardiographic variables are among the last remaining “new” (i.e., previously unused) markers to include in ED risk stratification and potentially improve upon the status quo.

Our study has several strengths. First, we prospectively tested our primary hypothesis and found TAPSE to be useful not only as a continuous variable but also at the binary cutoff we specified a priori from prior research.^17^ Due to a large effect size, we were able to detect a difference even with a slightly abbreviated sample size (on account of the COVID-19 pandemic) compared to our prespecified power analysis. TAPSE ≥ 17 mm, after adjusting for STRATIFY, predicted a further fourfold reduction in odds of adverse events which was greater than anticipated. Second, we used rigorous methods^38^ to adjudicate AHF diagnosis and outcomes, including multiple blinded raters and multiple redundant information sources for follow-up, and included follow-up methods designed to detect outcomes occurring outside our institutions (e.g., HIE data and telephone follow-up). Third, ED ultrasound examinations were performed at the point of care primarily by attending physician and resident investigators (NH, JL) without advanced fellowship training in POCUS, but measurements were nevertheless shown to have high ICC with blinded expert testamurs of the NBE (MF, RE) and one additional blinded offline reviewer (LG). This included measurements expected to be the most challenging for EPs, such as fwRVLS. Every patient screened in whom a POCecho was attempted had adequate images to measure TAPSE, compared to two patients in whom LVEF (a basic POCecho requirement for all EM residents) could not be obtained. Fourth, we measured a broad swath of echocardiographic variables far earlier in the treatment course than any prior studies of POC or formal echocardiography, while enrolling a broad mix of acuity. As shown in a previously published report from REED-AHF,^38^ measures of RVD like TAPSE and those of PH like PASP changed dramatically between the initial ED echocardiograms and even 24 h later. The next earliest enrolling AHF study evaluating right heart function for risk prediction^25^ included patients hours after hospital admission, meaning that we were able to observe RVD and PH that may otherwise have been missed. Finally, we used a novel approach with face validity to address a key risk of bias affecting all studies of ED risk prediction/stratification in AHF: i.e., the vast majority of AHF patients are admitted to the hospital, and admission could change risk compared to discharge. We approached this problem by tying our most important measures (ΔSensitivity [NRI_events_] and ΔSpecificity [NRI_nonevents_]) to risk thresholds more conservative than the actual outcome rates experienced by patients whom POCecho-blinded EPs chose to discharge. This was done to acknowledge the status quo: conservative disposition practices resulting in admission for 80%–90% of patients and < 50% of patients receiving an intervention requiring hospital admission (Table 1—new oxygen requirement, PPV/intubation, IV nitro, and/or higher total IV diuretic dose than allowed to be given as a single dose by ACC guidelines^61^). Concurrently, rather than seeking primarily to answer broad but less clinically actionable questions about prediction across the total spectrum of risk^60^ (e.g., whether TAPSE improves AUROC), we asked whether TAPSE could improve identification of patients on the low-risk end of the spectrum whom clinicians might then be able to consider for outpatient therapy (discharge or observation). This approach was specifically designed to match the critical need of an unnecessarily high admission rate in the United States (possibly by 50% or more^8^) with a clinically actionable goal: if an ED patient has no absolute indications for inpatient status (e.g., supplemental oxygen), adding TAPSE ≥ 17 mm to a STRATIFY risk score can increase the odds of appropriately identifying individuals with lower-than-average rates of adverse 30-day events to help facilitate safe outpatient management in an otherwise high-risk population.

## LIMITATIONS

Our study also has several limitations. First, despite the methods described to address the bias of high admission rates in AHF risk stratification literature, we cannot eliminate this bias entirely. The only way to do this completely would be to enforce mandatory EP disposition decisions in a randomized trial based on TAPSE and STRATIFY.

Second, our study was powered to detect whether TAPSE ≥ 17 mm could add predictive utility to the existing STRATIFY score, which is not the same as if we had refit a new model including all 13 individual variables in STRATIFY plus TAPSE (i.e. as the 14th). At the outcome rates observed, we would have required more than eight times the number of patients enrolled to have enough events to prevent overfitting on logistic regression fitting an entirely new CDI with as many variables^13^ as STRATIFY. Given the feasibility of obtaining POCechos early in the course and the novel nature of our hypothesis among ED studies, this was not seen as feasible and therefore not the goal of REED-AHF. Based on our current results a future study of that scope may now be justified, to derive a de novo CDI including prospectively collected TAPSE and/or other POCecho markers alongside traditional clinical variables.

Third, we had slightly less than 10 events per variable when considering both the STRATIFY score and a POCecho variable (i.e., eight events per variable). Our sample size was slightly abridged due to the COVID-19 pandemic as discussed, and prior research has shown that as low as five events per variable is usually sufficient to prevent overfitting.^67^ Moreover, the STRATIFY score actually performed better by AUROC in our sample (0.706 after bootstrap correction for bias/optimism) than in its original derivation,^8^ making it doubtful that our results are due to poor performance of our chosen confounder (STRATIFY) in the 16 events observed. Likewise, predicted bias/optimism in the TAPSE-adjusted model^59^ was overall low (AUROC optimism 0.026, with an observed AUROC 0.796 vs. 0.756 bias-corrected), suggesting a lower chance that a higher event count would have led to much worse performance in an external sample or if enrollment targets had been met. Nevertheless, we cannot say for certain how completion of the original enrollment target would have changed results, which in turn makes our results more hypothesis generating than overtly practice changing. Ultimately, as mentioned, a larger study than even our initial enrollment target will be needed to derive and then validate direct incorporation of TAPSE into a de novo CDI. A future study to externally validate our results here is also warranted, especially in view of this limitation.

Fourth, we did not directly test learning curves for TAPSE in EP hands (e.g., compared to current requirements for EM residency in POCecho), although ICC to experts was high and feasibility of EP-performed TAPSE for risk stratification has been demonstrated in prior studies of PE.^47,48^ No patients were excluded from the current study for images insufficient to grade TAPSE, but a future study to directly describe learning curves and pedagogy in EM learners would be helpful.

Fifth, the study was not designed to compare POCecho variables head to head, and the abridged enrollment likely decreased this power even further. Given this, we cannot say that other echocardiography variables such as LVEF and mitral inflow parameters are of low utility, but rather only that we had low power to detect a difference among various echocardiography parameters. Nevertheless, other measures all had lower variable importance (Figure 2) and aOR effect size (Figure S1) than TAPSE.

Sixth, our population is medically underserved, predominantly non-White, coming from only two hospitals (DRH, SGH) and two institutions (Wayne State and Michigan State University College of Osteopathic Medicine). Generalizability to different populations is therefore uncertain.

## CONCLUSIONS

Tricuspid annulus plane systolic excursion (TAPSE) significantly improved risk stratification compared to the validated STRATIFY clinical decision instrument. Most critically, TAPSE appears to improve detection of low-risk acute heart failure, even among patients misclassified as high risk. TAPSE, the TAPSE/PASP ratio, the TAPSE/RVDD ratio, and fwRVLS may all help increase the identification of low-risk acute heart failure patients in the ED who could be considered for outpatient treatment rather than hospital admission. Our hypothesis-generating results support a need to further define and evaluate point-of-care echocardiography’s role in acute heart failure risk stratification. Future studies should investigate the incorporation of point-of-care echocardiography variables, and particularly right-heart measures, into acute heart failure clinical decision instruments given their demonstrated potential here, the face validity for acute heart failure risk-stratification, and the status of echocardiography as one of the few accessible clinical parameters not already used to risk-stratify acute heart failure in the emergency department.

## Supplementary Material

Fs1

SUPINFO

Fs2

## Figures and Tables

**TABLE 1:** Selected clinical characteristics of the cohort

Characteristic	Overall, *N* = 84^a^	No 30-day events, *n* = 68^a^	≥1 event at 30days, *n* = 16^a^	*p*-value^b^
STRATIFY score for ED AHF				
Age (years)	62 (54–70)	61 (54–70)	65 (60–70)	0.8
BMI	28 (24–36)	30 (23–37)	27 (24–32)	0.8
Brain natriuretic peptide	1098 (568–1910)	1160 (500–1898)	688 (615–2151)	>0.9
Diastolic blood pressure (mm Hg)-arrival	90 (79–101)	89 (78–100)	94 (81–108)	0.5
Blood urea nitrogen	22 (17–36)	22 (17–33)	27 (22–37)	0.2
Sodium	138 (136–140)	139 (137–141)	136 (134–138)	0.001
Respiratory rate	20 (20–26)	20 (20–25)	22 (20–32)	0.4
SpO_2_ (%)-arrival	97 (95–98)	96 (95–98)	99 (96–100)	0.11
Troponin I (ng/mL)	0.07 (0.04–0.13)	0.07 (0.03–0.12)	0.09 (0.07–0.22)	0.068
History of dialysis	5 (6.0)	2 (2.9)	3 (19)	0.045
Any supplemental oxygen in ED	47 (56)	36 (53)	11 (69)	0.3
ACE inhibitor or ARB	32 (38)	27 (40)	5 (31)	0.5
Wide QRS on ECG	17 (20)	13 (19)	4 (25)	0.7
Total ED stratify score	215 (198–233)	212 (196–228)	233 (218–266)	0.009
Ranked primary outcome (0–5) at 30days				<0.001
0 = no 30-day event	68 (81)	68 (100)	0 (0)	
1 = AMI, PCI, and/or CABG	8 (9.5)	0 (0)	8 (50)	
2 = new or emergent dialysis	1 (1.2)	0 (0)	1 (6.2)	
3 = intubation	4 (4.8)	0 (0)	4 (25)	
4 = mechanical cardiac support	0 (0)	0 (0)	0 (0)	
5 = Death/CPR	3 (3.6)	0 (0)	3 (19)	
Other clinical characteristics^c^				
Systolic blood pressure (mm Hg)-arrival	153 (132–173)	152 (134–170)	156 (129–184)	0.7
Male sex	50 (60)	39 (57)	11 (69)	0.4
History of COPD, OSA, or OHS	55 (65)	43 (63)	12 (75)	0.4
History of pulmonary hypertension	27 (32)	23 (34)	4 (25)	0.5
IV furosemide (mg)				
ED	40 (40–40)	40 (40–40)	40 (20–40)	0.3
24 h	80 (40–80)	80 (40–80)	40 (40–80)	0.058
Total ED and hospital	120 (60–205)	120 (75–200)	180 (55–285)	0.3
Total ED and hospital furosemide > 200 mg^d^	21 (25)	14 (21)	7 (44)	0.1
Oxygen requirement in ED greater than baseline	39 (46)	30 (44)	9 (56)	0.4
ED NIPPV	15 (18)	12 (18)	3 (19)	>0.9
Intubated in ED	1 (1.2)	0 (0)	1 (6.2)	0.2
ED IV nitroglycerin	10 (12)	8 (12)	2 (12)	>0.9
ED disposition				
ICU	3 (3.6)	2 (2.9)	1 (6.2)	0.5
Observation	12 (14)	11 (16)	1 (6.2)	0.4
Discharged from ED	8 (9.5)	7 (10)	1 (6.2)	>0.9

Abbreviations: ACE, angiotensin-converting enzyme; AMI, acute myocardial infarction; ARB, angiotensin receptor blocker; BMI, body mass index; CABG, coronary artery bypass graft; ECG, electrocardiogram; ICU, intensive care unit; NIPPV, noninvasive positive pressure ventilation; PCI, percutaneous coronary intervention; SpO_2_, oxygen saturation.

a Data are reported as median (IQR) or *n* (%).

b Wilcoxon rank-sum test; Fisher’s exact test.

c See Harrison et al.^38^ for a more comprehensive description of the REED-AHF cohort, including additional home/ED medications, medical history, risk scores, other additional factors.

d 200 mg is the maximum IV furosemide allowed to be given as a single dose per the American College of Cardiology’s recommendations.^61^

**TABLE 2:** Echocardiographic variables obtained on point-of-care examination at ED arrival

POCecho variable	Overall, *N* = 84	Admitted to hospital, *n* = 76	Discharged from ED, *n* = 8	p-value, admit vs. discharge	No 30-day event, *n* = 68	≥1 event at 30 days, *n* = 16	p-value, event^a^ vs. no event
Right heart measures							
TAPSE (mm)	17 (13–23)	17 (13–23)	21 (17–22)	0.3	18 (14–23)	13 (12–16)	0.011
TAPSE/RVDD ratio (mm/mm)	3.73 (2.54–5.33)	3.70 (2.42–5.33)	4.32 (3.57–5.29)	0.4	4.09 (3.07–5.37)	2.73 (2.13–3.94)	0.035
TAPSE/PASP ratio (mm/mmHg)	0.37 (0.29–0.56)	0.35 (0.29–0.54)	0.49 (0.46–0.65)	0.03	0.39 (0.30–0.65)	0.29 (0.26–0.37)	0.011
PASP [RVSP] (mm Hg)	43 (33–50)	44 (35–51)	36 (30–43)	0.1	43 (33–49)	46 (37–53)	0.3
RV/LV diameter ratio	0.81 (0.71–0.95)	0.81 (0.71–0.95)	0.76 (0.65–0.92)	0.3	0.81 (0.69–0.91)	0.79 (0.73–1.01)	0.4
RVOT PW Doppler acceleration time (ms)	84 (67–104)	84 (67–104)	84 (75–98)	0.7	82 (68–104)	86 (64–101)	0.6
Pulmonary vascular resistance (Wood units)	3.47 (2.43–4.90)	3.47 (2.39–5.10)	3.08 (2.68–3.95)	0.6	3.41 (2.41–4.60)	4.20 (2.82–6.07)	0.2
RV FAC	0.33 (0.21–0.42)	0.32 (0.21–0.42)	0.37 (0.32–0.54)	0.14	0.34 (0.23–0.42)	0.28 (0.20–0.44)	0.4
fwRVLS	15 (11–21)	15 (11–21)	13 (10–20)	0.8	16 (11–21)	12 (7–15)	0.034
Left heart measures							
LVEF	28 (19–38)	29 (20–39)	20 (18–29)	0.14	28 (19–36)	34 (22–41)	0.4
E/A ratio	3.07 (1.48–4.16)	3.07 (1.48–4.16)	3.08 (1.53–3.88)	0.9	2.97 (1.49–4.32)	3.29 (1.51–4.10)	0.8
E/E’	18.5 (15.4–22.5)	18.7 (15.7–22.5)	16.4 (15.2–19.6)	0.6	18.5 (16.0–22.3)	18.7 (13.6–22.0)	0.6
E’ septal	0.04 (0.03–0.06)	0.04 (0.03–0.06)	0.05 (0.03–0.06)	>0.9	0.05 (0.03–0.06)	0.04 (0.04–0.05)	0.6
E’ lateral	0.06 (0.05–0.08)	0.06 (0.05–0.08)	0.06 (0.05–0.08)	0.7	0.06 (0.05–0.08)	0.06 (0.05–0.07)	0.3
LVGLS	6.8 (4.8–10.2)	6.9 (4.6–10.6)	6.7 (6.0–6.9)	0.8	6.9 (4.9–10.4)	6.7 (4.5–9.0)	0.4

*Note*: Fifteen echocardiographic variables were stratified by ED disposition (left) and occurrence of at least one event in the composite primary 30-day outcome^a^ (right). All echocardiography measures were collected at the point of care less than 1 h after, or any time before, first initiation of an AHF treatment in the ED (positive pressure ventilation, IV loop diuresis, IV or sublingual nitroglycerin). Only TAPSE, its ratio to RVDD and PASP, and fwRVLS were associated with the primary outcome. Only the TAPSE/PASP ratio differed between dispositions, as determined by the treating EP blinded to echocardiography. Wilcoxon rank-sum test used for unadjusted statistical comparisons.

Abbreviations: FAC, fractional area change; fwRVLS, free-wall right ventricular longitudinal strain; LV, left ventricle; LVEF, left ventricular ejection fraction; PASP, pulmonary artery systolic pressure; POCecho, point-of-care echocardiography; RVSP, right ventricular systolic pressure; PW, pulsed wave; RV, right ventricle; RVDD, right ventricular diastolic diameter; RVOT, right ventricular outflow tract; TAPSE, tricuspid annular plane systolic excursion.

a 30-day death, CPR, mechanical cardiac support, intubation, new or emergent dialysis, acute myocardial infarction, and/or coronary revascularization.

**TABLE 3:** Category-free net reclassification and receiver operating characteristic AUC for TAPSE and selected clinical comparators

		AUROC (validation/bias-corrected)	
Variable of Interest	Category-free net reclassification index (NRI_cont_) [95% CI]	STRATIFY + variable of interest (full models)	Difference compared to STRATIFY score only (reduced model, AUC of 0.706)
TAPSE (mm)	+0.63 [+0.08 to +1.18]	0.756	+0.050
History COPD	+0.02 [−0.52 to +0.56]	0.683	−0.023
History pulmonary hypertension	+0.18 [−0.30 to +0.66]	0.676	−0.030
GDMT adherence before ED	+0.26 [−0.05 to +0.56]	0.712	+0.006
ED arrival SBP	−0.04 [−0.58 to +0.50]	0.689	−0.017
Male sex	+0.23 [−0.28 to +0.74]	0.685	−0.021
IV furosemide in ED	+0.44 [−0.10 to +0.98]	0.714	+0.008
New or increased O_2_ requirement in ED	+0.19 [−0.33 to +0.71]	0.700	−0.006
ED NIPPV or intubation	−0.11 [−0.57 to +0.36]	0.690	−0.016
ED IV nitro	+0.21 [−0.20 to +0.62]	0.697	−0.009
ED disposition			
Discharge	+0.08 [−0.20 to +0.36]	0.725	+0.019
ICU	+0.22 [−0.19 to +0.64]	0.678	−0.028

*Note*: TAPSE improved net prediction/classification when added to the STRATIFY risk score (NRI_cont_ CI does not cross zero).^a^ Overall, the risk stratification benefits added to STRATIFY by TAPSE were not observed for EP standard-of-care risk stratification or other observed variables. Potential confounding comorbidities and treatment differences outside of the 13 variables already captured by STRATIFY failed to match or explain the TAPSE-related improvement in classification (all NRI_cont_ *p* > 0.05) and prediction (smaller change in ROC AUC compared to TAPSE). Likewise, the improvement in classification and prediction observed with TAPSE was not observed in the actual disposition decisions of EPs blinded to echocardiogram results.

Abbreviations: COPD, chronic obstructive pulmonary disease; GDMT, guideline-directed medical therapy for heart failure; ICU, intensive care unit; NIPPV, noninvasive positive pressure ventilation; PH, pulmonary hypertension; ROC AUC, receiver operating characteristic area under the curve.

a Primary outcome of the STRATIFY score = 30-day death, CPR, mechanical cardiac support, intubation, new or emergent dialysis, acute myocardial infarction, and/or coronary revascularization.
