# Supplementary material for: Point‐of‐care echocardiography of the right heart improves acute heart failure risk stratification for low‐risk patients: The REED‐AHF prospective study
Source: Acad Emerg Med. 2022 Sep 26;29(11):1306–19. doi: 10.1111/acem.14589 (PMC9671834; doi:10.1111/acem.14589)
Supplement: Supplementary file 1 — Figure S1 Figure S2 [file ACEM-29-1306-s001.zip › ACEM_14589_Odds Ratio Forest Plot v2.pdf]

Echo Variables - Continuous (Odds Ratios per IQR)

Echo Variable	OR (95% CI)	aOR (95%CI)
TAPSE	0.33 (0.13 - 0.82)	0.32 (0.12 - 0.87)
TAPSE/RVDD ratio	0.37 (0.15 - 0.91)	0.35 (0.13 - 0.94)
TAPSE/PASP ratio	0.39 (0.16 - 0.92)	0.38 (0.15 - 0.96)
PASP (RVSP)	1.45 (0.68 - 3.07)	1.5 (0.67 - 3.35)
RV/LV ratio	1.73 (0.69 - 4.38)	1.9 (0.72 - 5.05)
RVOT accel. time	0.76 (0.34 - 1.72)	0.65 (0.25 - 1.66)
PVR	1.22 (0.73 - 2.02)	1.25 (0.75 - 2.1)
RV FAC	0.48 (0.23 - 1.02)	0.41 (0.17 - 0.94)
fwRVLS	0.39 (0.14 - 1.05)	0.31 (0.11 - 0.9)
LVEF	1.39 (0.57 - 3.41)	1.34 (0.52 - 3.44)
E/a ratio	1.65 (0.16 - 17.1)	1.17 (0.62 - 2.21)
E/E’ ratio	1.54 (0.77 - 3.07)	1.33 (0.64 - 2.78)
E’ septal	0.67 (0.29 - 1.57)	0.84 (0.32 - 2.18)
E’ lateral	0.62 (0.27 - 1.39)	0.73 (0.29 - 1.87)
LVGLS	0.62 (0.27 - 1.46)	0.57 (0.23 - 1.43)

Unadjusted

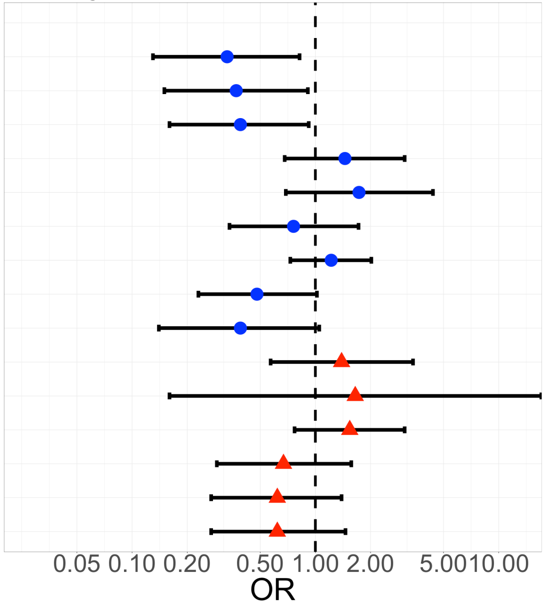

Adjusted for STRATIFY

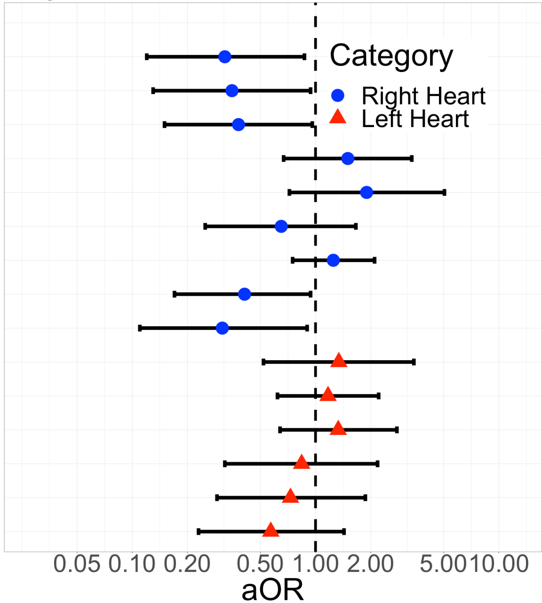

Echo Variables at Pre-specified Categorical Cuttoffs

Echo Variable	OR (95% CI)	aOR (95%CI)
TAPSE ≥17mm	0.22 (0.06 - 0.77)	0.24 (0.06 - 0.91)
TAPSE/RVEDD ratio ≥ 0.36 (mm/mm)	0.28 (0.09 - 0.93)	0.25 (0.07 - 0.9)
TAPSE/PASP ratio ≥ 0.29 mm/mmHg	0.21 (0.06 - 0.7)	0.22 (0.06 - 0.78)
PASP (RVSP) ≤ 40mmHg	0.8 (0.23 - 2.74)	0.68 (0.19 - 2.53)
RV/RV ratio < 1	0.31 (0.08 - 1.14)	0.24 (0.06 - 0.99)
RVOT accel. time > 90ms & no notching	0.7 (0.19 - 2.49)	0.55 (0.13 - 2.27)
PVR < 3 Wood units	0.92 (0.24 - 3.5)	0.94 (0.24 - 3.71)
RV FAC > 35%	0.4 (0.11 - 1.38)	0.32 (0.08 - 1.23)
fwRVLS > 17	0.31 (0.08 - 1.26)	0.28 (0.06 - 1.24)
LVEF > 40%	2.12 (0.61 - 7.38)	1.93 (0.5 - 7.5)
E/a < 2	0.87 (0.26 - 2.89)	0.87 (0.24 - 3.16)
E/E’ ≤ 14	1.32 (0.36 - 4.85)	1.78 (0.42 - 7.56)
E’ septal ≥ 7 cm/s	0.27 (0.03 - 2.32)	0.58 (0.06 - 5.64)
E’ lateral ≥ 9 cm/s	0.4 (0.05 - 3.52)	1.06 (0.1 - 11.34)
LVGLS > 5.1	0.49 (0.15 - 1.63)	0.47 (0.13 - 1.65)

Unadjusted

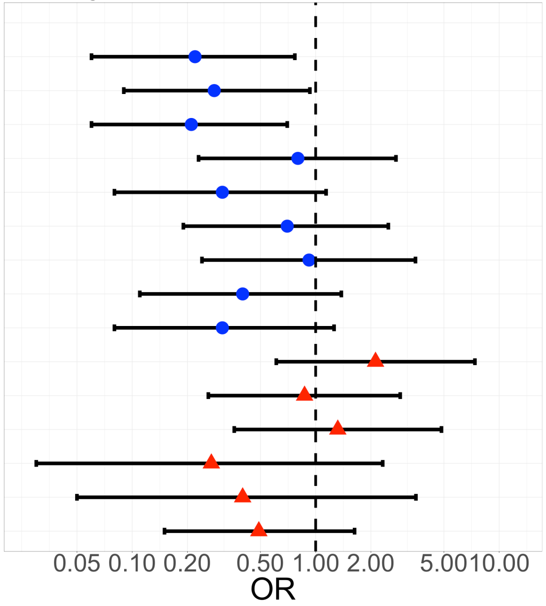

Adjusted for STRATIFY

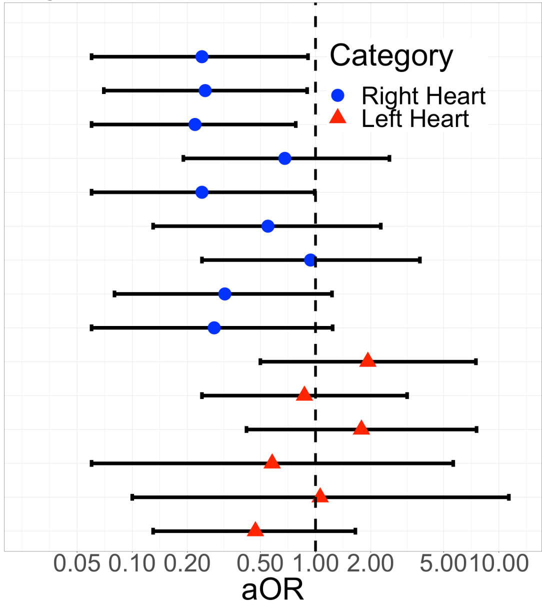

Outcome: Death, MCS, Intubation, New/Emergent Dialysis, and/or AMI/PCI/CABG
