# Supplementary material for: Point‐of‐care echocardiography of the right heart improves acute heart failure risk stratification for low‐risk patients: The REED‐AHF prospective study
Source: Acad Emerg Med. 2022 Sep 26;29(11):1306–19. doi: 10.1111/acem.14589 (PMC9671834; doi:10.1111/acem.14589)
Supplement: Supplementary file 1 — Figure S1 Figure S2 [file ACEM-29-1306-s001.zip › ACEM_14589_SUPPLEMENTARY FIGURE LEGENDS AND DISCUSSION.docx]

**SUPPLEMENTARY FIGURE LEGENDS AND DISCUSSION**

**Supplemental Figure S1: Odds Ratios for the Prediction of Serious Adverse Heart Failure Events By 9 Right-Heart and 6 Left-Heart Echocardiographic Variables, Before and After Adjustment for the STRATIFY Decision Instrument**

Top: POCecho measures as continuous variables. Bottom: POCecho variables at pre-specified binary cutoffs from the American Society of Echocardiography guidelines and/or prior literature (see methods). 95% Confidence Interval (95%CI) represented as horizontal bars, with bars not crossing 1 (dotted vertical line) indicating statistical significance (p<0.05). TAPSE and multiple other right heart measures (Blue) reached statistical significance, unlike any left-heart (Red) measures.

TAPSE = tricuspid annular plane systolic excursion; RVDD = right ventricular diastolic diameter; fwRVLS = free wall RV longitudinal strain; FAC = RV fractional area change; PASP = pulmonary artery systolic pressure; RV = right ventricle; LV = left ventricle; LVEF = left ventricular ejection fraction; PVR = pulmonary vascular resistance; LVGLS = LV global longitudinal strain; RVOT = right ventricular outflow tract; PW = pulsed wave.

**Supplemental Figure S2: Change in Sensitivity (NRI-Events) and Specificity (NRI-Nonevents) for 15 Different Echocardiographic Measures Added to STRATIFY vs. STRATIFY Alone, for Various Thresholds of 30-day Risk for Serious Adverse Heart Failure Events**

Categorical reclassification of patients experiencing an event* (NRI_events_ / Δ sensitivity / Δ TPR; red lines with 95% CI) or event-free (NRI_non-events_ / Δ specificity / Δ TNR; blue lines with 95% CI) are shown for the addition of each echocardiographic variable to the STRATIFY risk score. Interpretation is similar to Figure 1, but the reclassification metrics for TAPSE are shown alongside the other 14 echo measures examined. Risk cutpoints of predicted 30-day event rates (i.e. “miss rates”) were examined from 3% (the smallest risk predicted by STRATIFY) to 10%, where 8.3% represents the standard of care miss rate (see text). Thus, cutpoints of ≤8% represent a risk-stratification strategy more conservative than usual care (emergency physician gestalt). TAPSE, TAPSE/PASP, TAPSE/RVDD, fwRVLS, and RV FAC all showed significant improvement in classification of non-events (i.e. an increase in specificity compared to STRATIFY alone). No measure was found to change (improve or worsen) sensitivity compared to STRATIFY alone, likely because STRATIFY was already highly sensitive (100% sensitivity for each cutpoint below 6%) but poorly specific ( ≤ 24% specific depending on cutpoint) for predicting the primary outcome. This highlights the main limitation for clinical practice of STRATIFY: with an average 80-90% admission rate for AHF patients seen in the emergency department, STRATIFY’s high sensitivity and low specificity are unlikely to be helpful in identifying the estimated 50% of admission which are unnecessary (i.e. could have been discharged or placed in observation). From this, the added specificity observed when adding multiple right-heart measures to STRATIFY (without sacrificing sensitivity) suggests these echo markers could be useful in helping to identify low-risk patients and reduce unnecessary admissions.

*Serious adverse heart failure events at 30-days: Death/CPR, Intubation, MCS new/emergent dialysis, AMI/PCI/CABG. This is the composite outcome on which the STRATIFY score was derived and validated previously.

NRI = net reclassification index; CI = confidence interval; TNR = true negative rate = NRI_non-events_ = Δ specificity; TPR = true positive rate = NRI_events_ = Δ sensitivity; TAPSE = tricuspid annular plane systolic excursion; RVDD = right ventricular diastolic diameter; fwRVLS = free wall RV longitudinal strain; FAC = RV fractional area change; PASP = pulmonary artery systolic pressure; RV = right ventricle; LV = left ventricle; LVEF = left ventricular ejection fraction; PVR = pulmonary vascular resistance; LVGLS = LV global longitudinal strain; RVOT = right ventricular outflow tract; PW = pulsed wave; MCS = mechanical cardiac support; AMI = acute myocardial infarction; PCI = percutaneous coronary intervention; CABG = coronary artery bypass graft
