# Supplementary figures and images for: Point‐of‐care echocardiography of the right heart improves acute heart failure risk stratification for low‐risk patients: The REED‐AHF prospective study
Source: Acad Emerg Med. 2022 Sep 26;29(11):1306–19. doi: 10.1111/acem.14589 (PMC9671834; doi:10.1111/acem.14589)

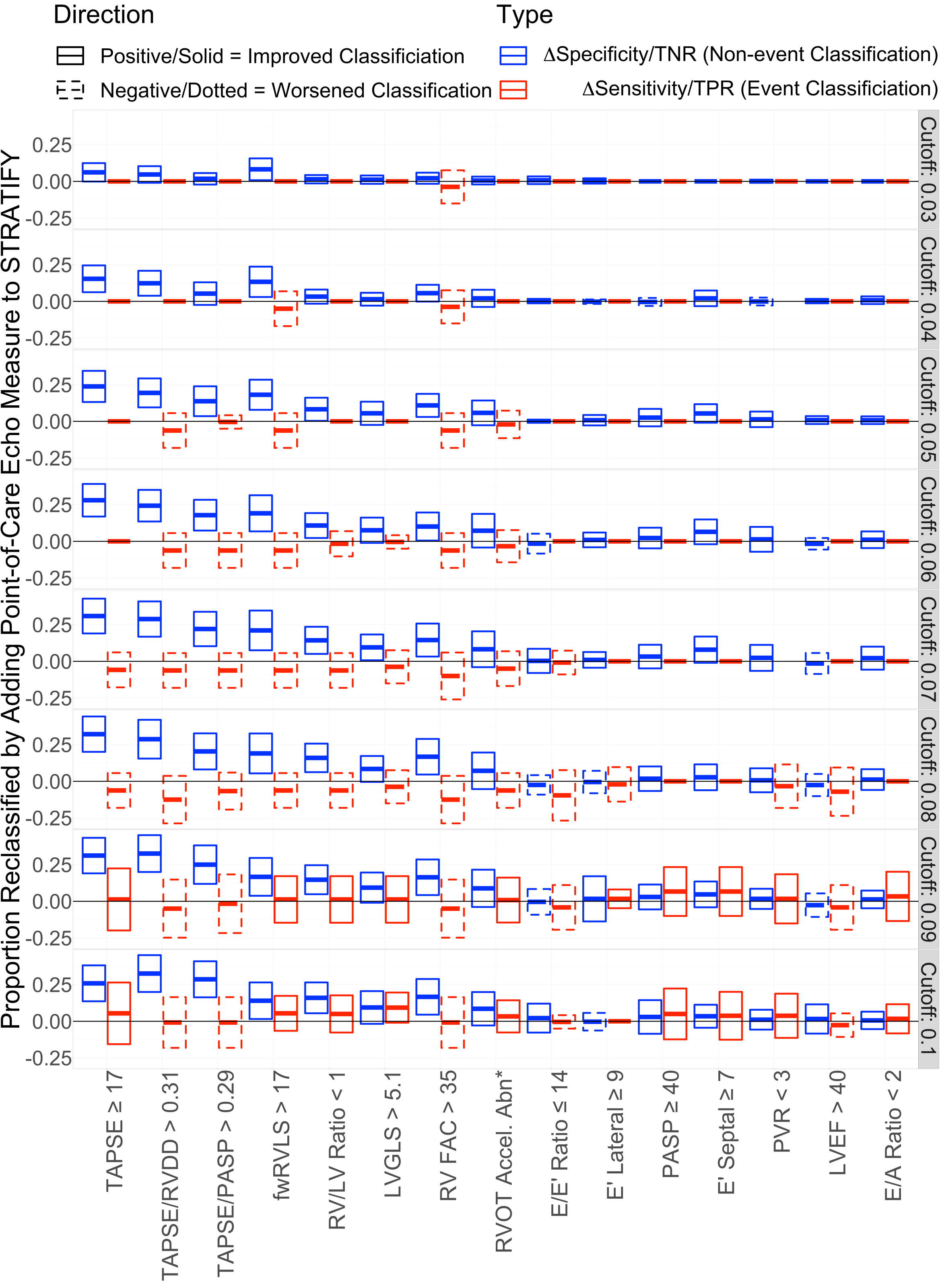

Supplement: Supplementary file 1 — Figure S1 Figure S2 [file ACEM-29-1306-s001.zip › ACEM_14589_Reclass Figure v2.pdf]
